# QSAR-Based Models for Designing Quinazoline/Imidazothiazoles/Pyrazolopyrimidines Based Inhibitors against Wild and Mutant EGFR

**DOI:** 10.1371/journal.pone.0101079

**Published:** 2014-07-03

**Authors:** Jagat Singh Chauhan, Sandeep Kumar Dhanda, Deepak Singla, Open Source Drug Discovery Consortium, Subhash M. Agarwal, Gajendra P. S. Raghava

**Affiliations:** 1 Bioinformatics Centre, Institute of Microbial Technology (CSIR), Chandigarh, India; 2 The Open Source Drug Discovery (OSDD) Consortium, Council of Scientific and Industrial Research, Anusandhan Bhavan, Delhi, India; 3 Bioinformatics Division, Institute of Cytology and Preventive Oncology, Noida, India; Huazhong University of Science and Technology, China

## Abstract

Overexpression of EGFR is responsible for causing a number of cancers, including lung cancer as it activates various downstream signaling pathways. Thus, it is important to control EGFR function in order to treat the cancer patients. It is well established that inhibiting ATP binding within the EGFR kinase domain regulates its function. The existing quinazoline derivative based drugs used for treating lung cancer that inhibits the wild type of EGFR. In this study, we have made a systematic attempt to develop QSAR models for designing quinazoline derivatives that could inhibit wild EGFR and imidazothiazoles/pyrazolopyrimidines derivatives against mutant EGFR. In this study, three types of prediction methods have been developed to design inhibitors against EGFR (wild, mutant and both). First, we developed models for predicting inhibitors against wild type EGFR by training and testing on dataset containing 128 quinazoline based inhibitors. This dataset was divided into two subsets called wild_train and wild_valid containing 103 and 25 inhibitors respectively. The models were trained and tested on wild_train dataset while performance was evaluated on the wild_valid called validation dataset. We achieved a maximum correlation between predicted and experimentally determined inhibition (IC_50_) of 0.90 on validation dataset. Secondly, we developed models for predicting inhibitors against mutant EGFR (L858R) on mutant_train, and mutant_valid dataset and achieved a maximum correlation between 0.834 to 0.850 on these datasets. Finally, an integrated hybrid model has been developed on a dataset containing wild and mutant inhibitors and got maximum correlation between 0.761 to 0.850 on different datasets. In order to promote open source drug discovery, we developed a webserver for designing inhibitors against wild and mutant EGFR along with providing standalone (http://osddlinux.osdd.net/) and Galaxy (http://osddlinux.osdd.net:8001) version of software. We hope our webserver (http://crdd.osdd.net/oscadd/ntegfr/) will play a vital role in designing new anticancer drugs.

## Introduction

The protein kinase is the largest known family within the human genome that contains more than 500 genes. These kinases play a vital role in signal transduction through phosphorylation mechanism, as they catalyze the transfer of phosphate from ATP to a hydroxyl group of serine, threonine or tyrosine of target proteins. Protein kinases are thus ATP binding proteins that exhibit highly conserved nature [1], [2]. As a result any functional deregulation of these enzymes results in disease states such as cancer, diabetes, inflammation, cardiovascular disease, neurological disorders, etc. Therefore, they have emerged as an important class of drug targets in drug discovery process. Imatinib, Nilotinib and Dasatinib are some of the drugs that were designed based on ATP binding sites of kinase target proteins [3]. To date, 11 kinase inhibitors have been approved by FDA for cancer treatment and other 80 kinase inhibitors are in clinical trial [4].

Epidermal growth factor receptor (EGFR) is a cell surface growth factor receptor kinase that has been involved in different types of cancers. EGFR is overexpressed in a number of cancers, including breast and lung cancer [5]–[10]. Gefitinib is a highly selective EGFR tyrosine kinase inhibitor that binds competitively to the ATP binding site [11] and is being used for treating lung cancer [9]. Erlotinib and Lapatinib are also other EGFR based inhibitors [12]. Basically, there are two main classes of EGFR inhibitors: quinazoline and pyrimidine derivatives. It has been also established that mutations in the tyrosine kinase domain of EGFR is responsible for causing Non small cell lung carcinoma (NSCLC) [13]. One of the most common oncogenic mutations is L858R, which accounts for approximately 41% of all activated mutations [14]. This mutation in the EGFR activates the kinase by disrupting auto-inhibitory interactions leading to a ligand-independent activation of TK activity and thus causes cancer. These mutations thus alter the kinase domain and they therefore represent distinct targets for inhibitor development. The crystal structure of the mutant L858R kinase in complex with inhibitors has been already experimentally determined that can play vital role in structure-based drug discovery [15].

Computational prediction of inhibitory activity and designing inhibitors using structural Bioinformatics analysis can be useful in accelerating drug development in the field of cancer. In the past, various methods have been developed mainly based on QSAR (quantitative structure−activity relationship) and molecular docking. Though models have been developed against EGFR/CDK [16]–[22], but none of them is available for public use. In the present study, we first develop models for predicting inhibitors against wild type EGFR. These models were trained, tested and validated on 128 anti-EGFR quinazoline derivatives that used in previous studies [16], [23], [24]. Secondly, we develop models for predicting imidazothiazoles and pyrazolopyrimidines based derivatives against mutant EGFR (L858R). Thirdly, we develop a hybrid model for predicting inhibitors against both wild and mutant EGFR. In order to facilitate drug discovery and serve the scientific community, we have implemented these models in the form of a webserver called ntEGFR.

## Methods

### Datasets

**Figure 1:**
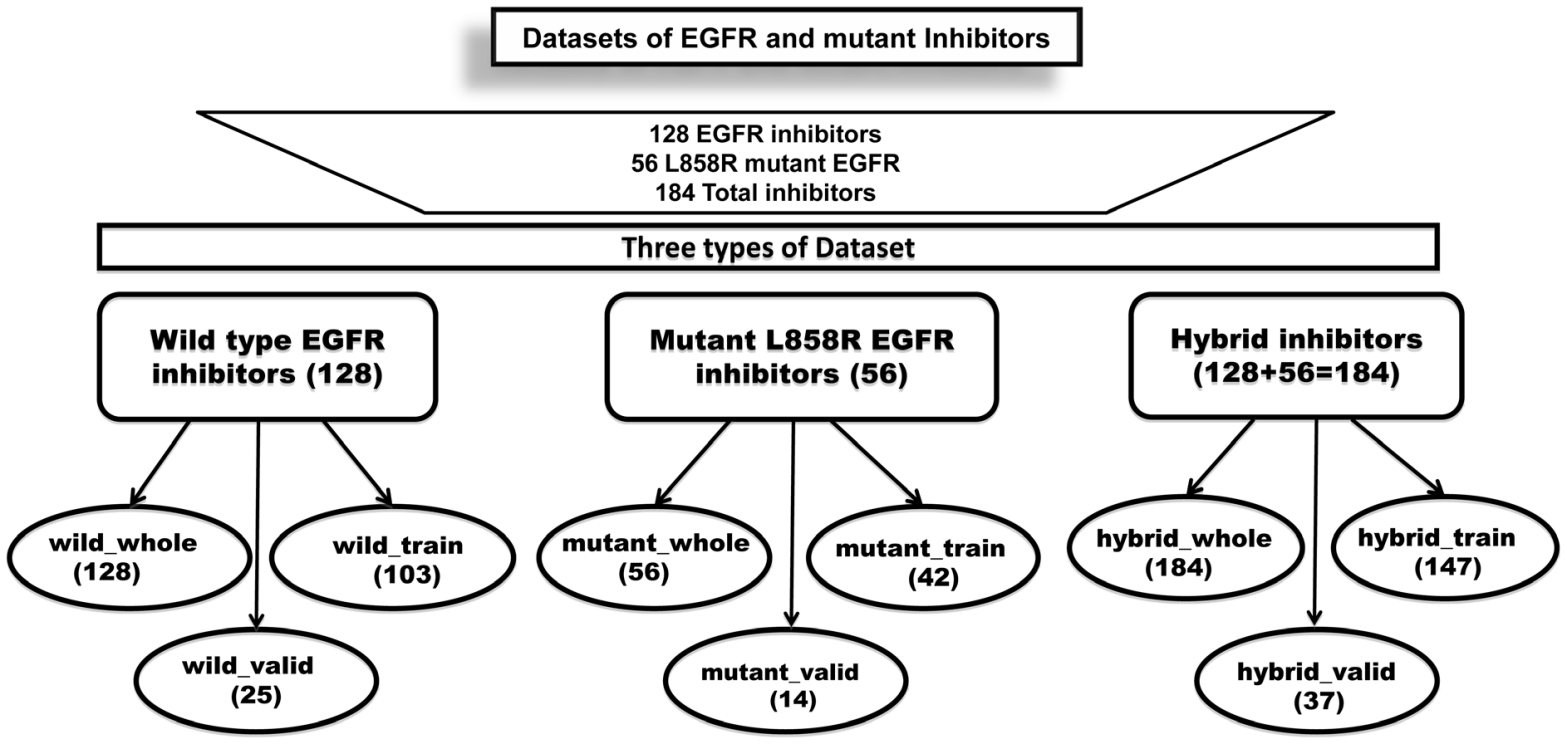
Flow chart showing training and validating datasets used in developing prediction models.

Creation of standard datasets for training, testing and validating models is one of the important parts of any in silico methods. In this study, we develop three types of models for predicting inhibitors against wild, mutant and both types of EGFR. Thus, we created three types of datasets for each type of model as described below (Figure 1).

#### (i) Datasets for wild type EGFR

In order to develop models against wild type of EGFR, we collected experimentally validated 128 anti-EGFR quinazoline derivative or quinazoline based inhibitors from the literature [16]. In this study, we called these inhibitors as wild type inhibitors and datasets consisting of all 128 inhibitors is called wild_whole dataset. In order to provide an unbiased evaluation of our models, we randomly divide our dataset into training (80% inhibitors) and validation (20% inhibitors) dataset. In summary, we created three datasets called wild_whole, wild_train and wild_valid which contains 128, 103 and 25 inhibitors respectively.

#### (ii) Datasets for mutant type EGFR

In addition to inhibitors reported against wild type of EGFR, we also collected 56 imidazothiazoles and pyrazolopyrimidines derivatives based inhibitors against mutant L858R EGFR with their inhibition constant value (IC_50_) from literature [25]–[27]. These 56 anti-EGFR mutant inhibitors were called as mutant type inhibitors and dataset consisting of these inhibitors was called mutant_whole dataset. Similar to above wild datasets, we created three datasets called mutant_whole, mutant_train and mutant_valid consisting of 56, 42 (80%) and 14 (20%) inhibitors respectively.

#### (iii) Hybrid datasets

In order to develop models for predicting inhibitor against both wild and mutant EGFR, we created a combined or hybrid dataset which consists of 184 inhibitors (128 wild + 56 mutant). This dataset was also divided into three datasets called hybrid_whole, hybrid_train and hybrid_valid consisting of 184, 147 and 37 inhibitors respectively.

### Biological activity

The inhibitory activities of all inhibitors are shown in pIC_50_ ( = −log (IC_50_)) values, where IC_50_ (nM) represents the concentration of compounds that produces 50% inhibition of the kinase activity. The training dataset was used for training the model and validation dataset was used for checking the performance and evaluating the prediction performance of trained model.

### Structure optimization

All the chemical structures were drawn using ChemDraw software (v.9.0). Vlife software (http://www.vlifesciences.com/) was used for optimization and checking for distorted and unrealistic bond angles and bond lengths. Energy minimization of all chemical structures was performed using molecular mechanism force field (MMFF94s) within Vlife software.

### Descriptors calculation

**Figure 2:**
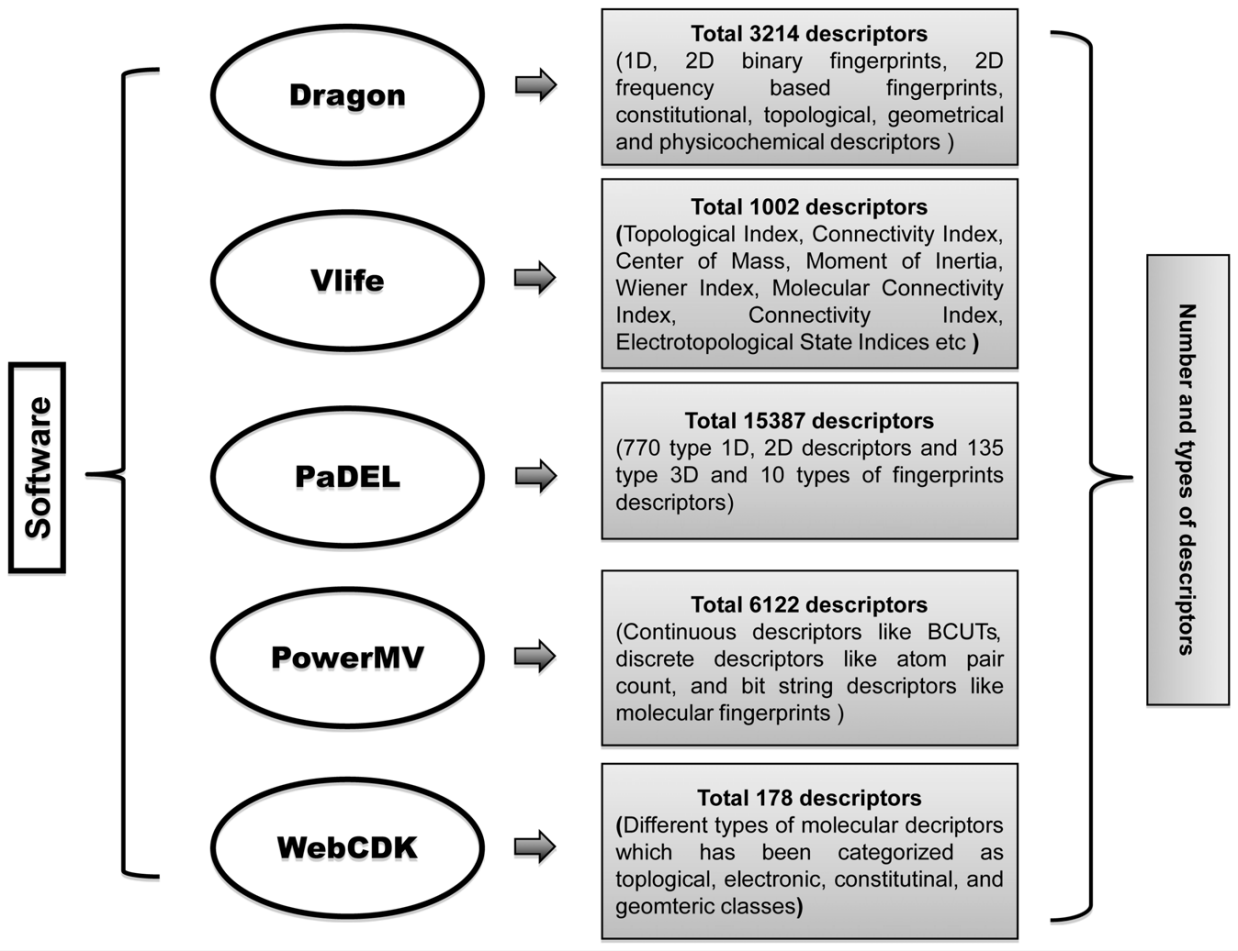
A diagram demonstrating list of softwares used for computing chemical descriptors and types of different descriptors.

In this study, we have computed all types of chemical descriptors, e.g. constitutional, topological, geometrical and physicochemical descriptors. We calculated descriptors of wild types inhibitors using Dragon (v.1.4) (http://www.talete.mi.it), Vlife, WebCDK, PaDEL [28] and PowerMV software while for mutant EGFR inhibitors descriptors were calculated using Vlife and PaDEL software packages (Figure 2).

### Receptor-ligand Preparation for docking

Protein (EGFR kinase, PDB ID: 1M17 and L858R mutant EGFR, PDB ID: 2ITZ) [15], [29] and ligand (EGFR inhibitor, Erlotinib and IRESSA) preparation was performed using the AutoDock 4.2 tool [30], which involved the addition of hydrogen atoms, computing charges, merging non-polar hydrogen atoms and defining AD4 atom types to ensure that atom conformed to the AutoDock atom types. A grid was defined using an Autogrid feature of the software and docking conformation search was done using a genetic algorithm (GA) procedure. Here, we set the grid point spacing of 0.375 Å with the default volume of 40×40×40 Å.

### Docking based energy descriptors

The AutoDock 4.2 tool computes seven types of energy values (i) Estimated free energy of binding (E_FreeBind_), (ii) Final Intermolecular Energy (EI_nterMol_), (iii) vdW + Hbond + desolv Energy (E_VHD_), (iv) Electrostatic Energy (E_Elec_), (v) Final Total Internal Energy (E_FToT_), (vi) Torsional Free Energy (E_Tors_) and (vii) Unbound System's Energy (E_Unb_). These descriptors were used independently to develop the model.

### Feature selection

As the selection of best and highly significant descriptors is crucial for QSAR modeling, feature selection was carried out to eliminate highly correlated descriptors, multicollinearity and remove useless descriptors. Thus, the descriptors with zero values were excluded followed by removal of highly correlated descriptors (cut-off value of 0.9). Subsequently, we used CfsSubsetEval module and F-stepping remove-one approach implemented in Weka for the significant descriptor selection. CfsSubsetEval evaluator is one of the most important feature selections pre-processing steps in the pattern classification, data mining, machine learning to remove non-significant feature [31], [32]. In F-stepping remove-one method, each input descriptor was removed one-by-one from the set of n descriptors followed by QSAR modeling using the remaining n-1 descriptors. If on removing the descriptor the correlation value decreased, the particular descriptor was permanently removed from the analysis. These cycles were repeated until no further improvement in the correlation values was observed and stopped if n-1 removal resulted in reduction of correlation values.

### QSAR models using Machine learning techniques

We used machine learning techniques for developing QSAR models for predicting inhibitors against wild or mutant EGFR. In this study, we developed models using support vector machine. Following software package were used to implement SVM.

### SVM models using SVM^light^

In this study, SVM based QSAR models has been implemented using software package SVM^light^ [33]. SVM^light^ is a user friendly software that is available free for acadamic use from following web site. http://www.cs.cornell.edu/People/ti/svm_light. In the past, this package has been used successfully in numreous studies for developing SVM based classification or regression models [34], [35]. The SVM^light^ pacakges have number of features that includes fast optimization, tuning of major kernels (e.g. linear, polynomial, radial basis function) or any user-defined kernel [33], [36]. In this study, SVM is implemented to solve regression problem.

### SVM models using SMOreg

In this study, we have used SMOreg (weka.classifiers.functions.SMOreg) for developing regression model. SMOreg module of Weka allows to implements support vector machine for regression with arbitrary kernel functions. The SMO algorithm transforms nominal attributes into binary form and it replaces all missing values globally. This algorithm have number of features that includes fast learning and better scaling properties [37]–[39].

### Evaluation of prediction model

The fitness and the statistical significance of the models developed in this study was assessed using the statistical parameters such as R, R^2^, MAE and RMSE.

**(i) Perasion's correation cofficient.** We compute correlation (R) between predicted and actual (experimentally determined) efficacy (IC_50_) of molecules using the following equation.
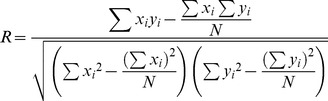
(i)


**(ii) Coefficient of determination (R^2^).** In addition to correlation coefficient R, we also compute coefficient of determination (R^2^). It is a statistical measure of how well the regression line approximates the real data points. An R^2^ of 1.0 indicates that the regression line perfectly fits the data. We used following formula for calculating R^2^.
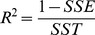
(ii)(where, SSE = sum of squared errors, SST = Total sum of squares)

**(iii) Mean Absolute Error.** In order to measure error between predicted and actual efficacy of molecules, we compute mean absolute error (MAE) using following formula.
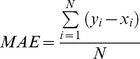
(iii)


**(iv) Root Mean Square Error.** In addition to MAE, we also compute root mean square error (RMSE) which is a frequently used statistical measure of the differences between values predicted by a model and the values actually observed. RMSE is a measure of predictive power of the model.



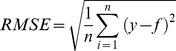
(iv)


## Results

### Fragment based analysis of datasets

As there are different kinds of molecules targeting the wild EGFR compared to mutant EGFR we have characterize the fragments that may be responsible for the biological activity. For the purpose, we have calculated the frequency of fragment occurrence in these two different datasets using the formula:
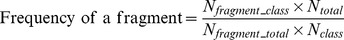
(v)Where N_fragment_class_ is the number of fragments present in that class (wild/mutant); N_total_ is the total number of molecules studied (wild + mutant); N_fragment_total_ is the total number of fragments in all molecules (wild + mutant); N_class_ is the number of molecules in that class (wild/mutant).

We observed that PubchemFP153, PubchemFP152, PubchemFP150, MACCSFP62, FP794, FP669, FP666, FP660, FP659, FP52, ExtFP895, ExtFP404, ExtFP370 and ExtFP318 descriptors were present in all mutant EGFR inhibitors however, were absent in all wild type EGFR inhibitors. On the other hand, the some fragments are more frequently present in the wild type EGFR (Table S1 in File S1). These fragments would be important in designing the inhibitors targeting only the wild EGFR. We have also extracted the fragments that were common among the inhibitors of EGFR in wild and mutant forms. Detail description about these fragments or chemical descriptors are given in Table S2 in File S1.

### Model for predicting inhibitors against wild type EGFR

**Figure 3:**
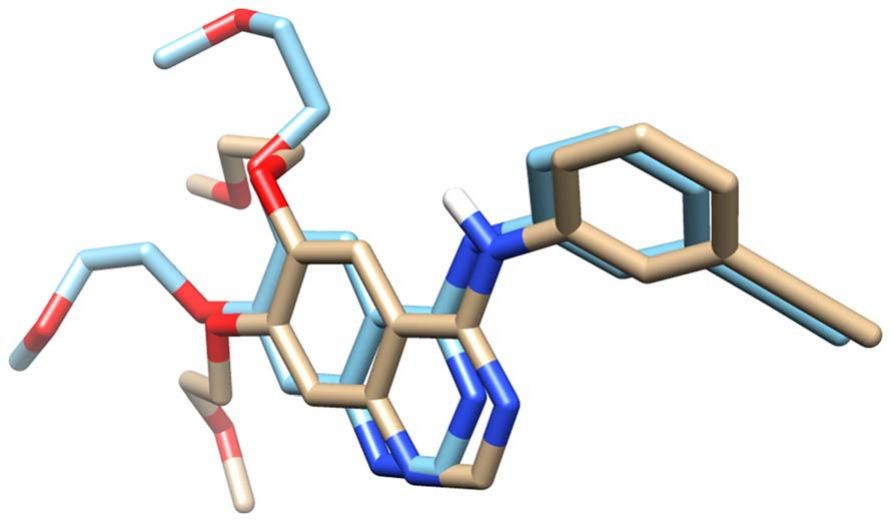
The superimposed structure of docked substrate over crystal structure conformation. Our docked structure is overlaid on the top of Erlotinib bound EGFR cristal stucture (PDB ID: 1M17).

In this study, we have trained, developed, tested and evaluated models using 128 quinazoline EGFR inhibitors whose inhibitory activity have been determined experimentally. QSAR models develoed using SMOreg (SVM for regression), a module of Weka package.We computed 3214, 1002, 15387, 178 and 6122 descriptors for 128 EGFR inhibitors using following packages; Dragon, Vlife, PaDEL, WebCDK and PowerMV respectively. In order to avoid over optimization, we restrict the number of descriptors to less than one fourth of total chemical compounds. We reduced the number of descriptors by removing irrelevant, duplicate and highly correlated descriptors. We have also used structure based approach along with ligand based approach as the high resolution target structure was available. Therefore, we carried out molecular docking for the 128 experimentally known EGFR inhibitors at the active binding site of PDB: 1M17 [29]. Erlotinib, the known EGFR inhibitor that has been co-crystallized was redocked into the active site of the EGFR kinase and 10 possible poses were generated. On visual examination of the ligand-EGFR complex we found that residue numbers 721, 764, 766, 769, and 831 are surrounding the active site while the residue Met769 is directly involved in hydrogen bonding with the inhibitor. The calculated RMSD between crystal and docked structure of best docked pose was 1.69 Å (Figure 3), which validated the docking protocol and thus for all the 128 inhibitors the same grid definitions were used.

#### (i) Performance of models on wild_whole datasets

We calculated seven docking energies based descriptors using Autodock and observed that descriptors namely E_FreeBind_, EI_nterMol_, E_VHD_, show pairwise correlation of more than 0.60. To obtain significant and non-correlated descriptors we filtered out these from seven descriptors for further analysis (Table S3 in File S1). We also observed that E_VHD_, EI_nterMol_, E_Tors_ and E_Unb_ show higher pairwise correlation coefficient values with respect to *IC*_50_ values. We then developed a model using seven docking energies based descriptors and achieved a correlation (R) of 0.27. Finally, we developed a model using 26, 15, 13, 32 and 24 non-correlated molecular descriptors of the Dragon, Vlife, PaDEL, WebCDK and PowerMV and thus we achieved correlation value (R and R^2^) in the range of 0.83/0.79, 0.80/0.77, 0.89/0.83, 0.77/0.73 and 0.81/0.78 respectively (Table 1).

**Table 1:** SMOreg based Performance of QSAR models based on selected descriptors of 128 wild EGFR inhibitors.

Descriptors	R	R^2^	MAE	RMSE
Vlife	0.801	0.773	0.513	0.692
Vlife+Dock energy	0.813	0.770	0.507	0.673
Dragon	0.835	0.791	0.456	0.640
Dragon+Dock energy	0.841	0796	0.444	0.624
WebCDK	0.773	0.734	0.570	0.730
WebCDK+Dock energy	0.777	0.734	0.557	0.723
PaDEL	0.891	0.835	0.438	0.567
PaDEL+Dock energy	0.892	0.836	0.425	0.546
PowerMV	0.811	0.786	0.531	0.677
PowerMV+Dock energy	0.815	0.787	0.529	0.667
Hybrid	0.911	0.843	0.371	0.497
Hybrid+Dock energy	0.921	0.847	0.349	0.450

**Figure 4:**
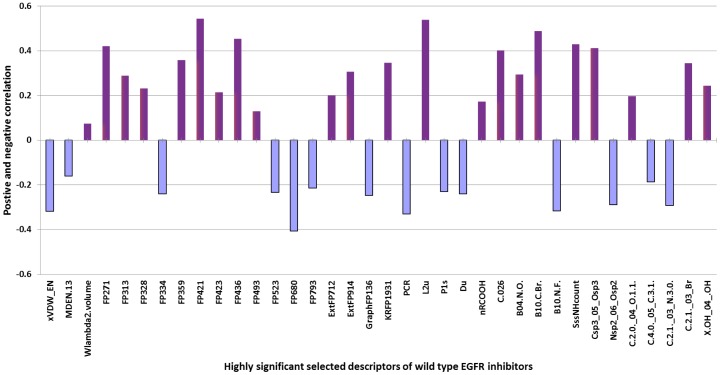
List of descriptors showing positive and negative correlation with the wild EGFR inhibitory activity.

We observe that models developed using PaDEL descriptors perform better than models developed using other software packages. We developed hybrid models that use descriptors generated by each of the five software packages along with autodock generated descriptors and achieve a correlation of 0.84, 0.81, 0.89, 078 and 0.82 between predicted and experimental IC_50_ value for Dragon, Vlife, PaDEL, WebCDK and PowerMV hybrid models respectively (Table 1). We also observed that 36 descriptors of Dragon, Vlife, WebCDK, PaDEL and PowerMV are highly significant, so we developed a model using these combined descriptors and achieved correlation (R) 0.91 and coefficient of determination (R^2^) 0.843 (Table 1). These selected descriptors either showed a positive correlation (FP271, FP313, FP359, FP421, FP436, ExtFP914, KRFP1931, L2u c.026, B04.N.O.,B10.C.Br, SssNHcount, Csp3_05_Osp3 and C.2.1._03_Br) where in the values of descriptor is directly proportional to inhibitory activity or negative correlation (xVDW_EN, FP334, FP680, GraphFP136, PCR, Nsp2_06_Osp2 and C.2.1._03_N3.0) where in the value of descriptors are inversely proportional to the inhibitory activity (Figure 4). Description of selected descriptors is given in Table S2 in File S1.

**Figure 5:**
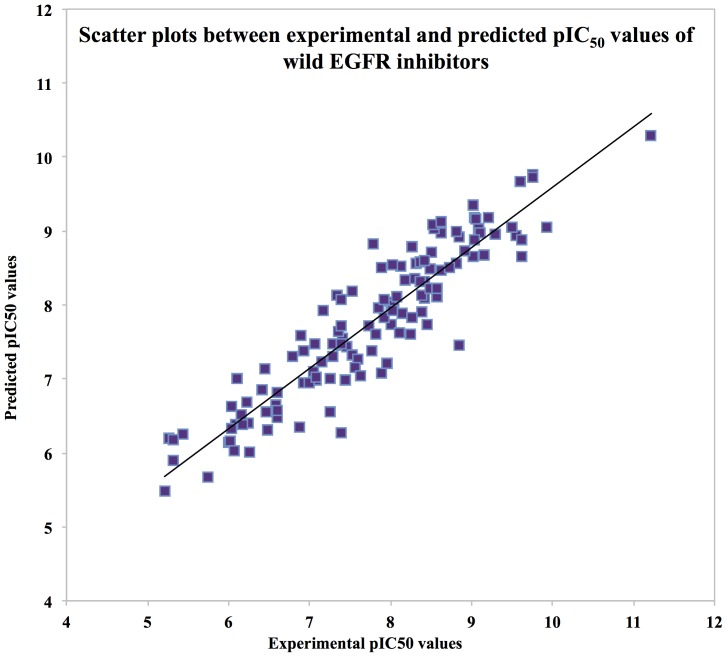
Scatter plots between experimental versus predicted pIC50 values of wild types EGFR inhibitors.

Finally we integrated all the 36 descriptors generated from each of the five software along with autodock descriptors to generate a final hybrid model that exhibits maximum correlation of 0.921 with R^2^ = 0.847 and MAE = 0.349 (Figure 5) (Table 1).

Here, we observed that the performance of hybrid model is slightly enhanced in comparison to the simple model. Thus, in this study, we have integrated two *in silico* techniques: QSAR and molecular docking by using docking generated energy-based descriptors for building the proposed models. As anticipated, the performance of hybrid model was better (R = 0.921 with R^2^ = 0.847 and MAE = 0.349) than the single models (Table 1).

#### (ii) Performance of model on training datasets

In this step we extracted 103 molecules for training from wild_whole dataset. In the next step we developed five fold cross-validation based model on 103 wild_train dataset and achieved a maximum correlation of 0.892 with MAE 0.392 and RMSE 0.514 (Table 2).

**Table 2:** Performance of SMOreg based models developing for predicting inhibitors against wild, mutant and hybrid EGFR on the training and validation data set on PaDEL descriptors.

Inhibitors	Descriptors	R	R^2^	MAE	RMSE
Wild EGFR	wild_whole (128 inhibitors)	0.891	0.835	0.438	0.567
	wild_train (103 inhibitors)	0.892	0.841	0.392	0.514
	wild_valid (25 inhibitors)	0.901	0.839	0.347	0.486
Mutant EGFR (L858R)	mutant_whole (56 inhibitors)	0.834	0.710	0.413	0.524
	mutant_train (42 inhibitors)	0.846	0.721	0.386	0.501
	mutant_valid(14 inhibitors)	0.850	0.745	0.368	0.467
Hybrid	hybrid_whole(184 inhibitors)	0.833	0.731	0.491	0.636
	hybrid_train(147 inhibitors)	0.850	0.723	0.464	0.628
	hybrid_valid (37 inhibitors)	0.761	0.623	0.617	0.724

#### (iii) Performance of model on validation dataset

In this method we have extracted 25 molecules (validation set) from 128 EGFR inhibitors to evaluate the performance of training model. Next we have checked the performance of wild_training inhibitors based model on the validation dataset and achieved a correlation (R)/coefficient of determination (R^2^) of 0.90/0.83 with MAE 0.34 and RMSE 0.48 (Table 2). Here, we also observed that the freely available PaDEl software performs comparable with commercial tools like Vlife and Dragon and therefore during mutant EGFR and hybrid model development only PaDEL software has been used.

### Evaluation of models developed on mutant EGFR inhibitors

Firstly, we used AutoDock for molecular docking of inhibitors in mutant EGFR. The co-crystallized ligand (IRESSA) was first extracted from the catalytic site of L858R EGFR mutant (PDB ID: 2ITZ) and re-docked to calculate the root mean square difference (RMSD) between the top docking pose and original crystallographic geometry. The calculated RMSD between crystal structure and docked structure of best-docked pose was 1.53 Å, which validated the docking protocol and thus for all the 56 inhibitors (imidazothiazoles and pyrazolopyrimidines derivatives) the same grid definitions were used. On visual examination of the ligand-EGFR complex we found that the residue Thr790 is directly involved in hydrogen bonding with the inhibitor. Secondly, we calculated seven docking energies based descriptors and observed that the three descriptors namely E_FreeBind_ (Free binding energy), EI_nterMol_ (Intermolecular energy) and E_VHD_ (vdW+Hbond+desolv Energy) showed a pairwise correlation more than 0.80 (Table S4 in File S1). So, we removed EInterMol as it has correlation close to 0.90 with E_VHD_. We then use the remaining six descriptors for generating the model and achieved a correlation of (R) = 0.445. Thirdly, using F-stepping remove one approach we eliminated each of the six descriptors from the set however, we did not observe any improvement in the correlation value of the model. We also observed that E_VHD_, EI_nterMol_, E_FToT_ and E_Unb_ show higher pairwise correlation coefficient values with respect to *IC*_50_ values.

#### (i) Performance of model on mutant_whole dataset

**Figure 6:**
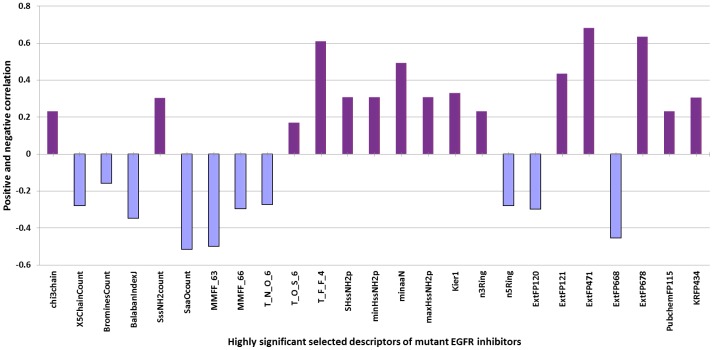
Positive and negative correlation of selected highly significant descriptors of mutant EGFR inhibitors.

First we calculate 15388 chemical descriptors of 56 L858R mutant EGFR inhibitors using PaDEL. After removing correlated descriptors and feature selection, we obtained 13 significant descriptors from a collection of 15388 descriptors, which have been used to generate models (Table 3). Some of these selected descriptors that exhibit high positive correlation, i.e. values of descriptor is directly proportional to inhibitory activity, are ExtFP471 (0.67), ExtFP678 (0.63), T_F_F_4 (0.62), minaaN (0.51) and ExtFP121 (0.50) whereas the other that show negative correlation, i.e. value of descriptors is inversely proportional to inhibitory activity, are SaaOcount (0.56), MMFF_63 (0.52) and ExtFP668 (0.51) (Figure 6). We then developed model via SMOreg technique using these 14 selected non-correlated highly significant descriptors of PaDEL and achieved a correlation coefficient (R)/coefficient of determination (R^2^) 0.8348/0.710 respectively. Thereafter, the significant descriptors selected from PaDEL were combined with docking based descriptors and hybrid models were developed having correlation (R)/coefficient of determination (R^2^) of 0.8412/0.7289 (Table 3).

**Table 3:** The SMOreg based performance of QSAR models developed using selected descriptors calculated from mutant_whole datasets.

Descriptors	No. of descriptors	R	R^2^	MAE	RMSE
Docking Energy	6	0.445	0.315	0.676	0.856
PaDEL descriptors	14	0.834	0.710	0.413	0.524
PaDELdescriptors+Docking Energy	20	0.841	0.728	0.398	0.517

#### (ii) Performance of model on training datasets (mutant_train)

In this step we extract 42 molecules for training (mutant_train) and developed a prediction model using five fold cross-validation and achieved a maximum correlation coefficient (R)/coefficient of determination (R^2^) of 0.8462/0.7214 with MAE 0.386 and RMSE 0.501 (Table 2).

#### (iii) Performance of hybrid model on validation dataset (mutant_valid)

We choose 14 inhibitors from different chemical cluster groups, which were not included in the training dataset. Next, we checked the performance of ligand and structure based model on the selected mutant EGFR inhibitors belonging to validation dataset and achieved a correlation (R)/coefficient of determination (R^2^) of 0.85/0.74 with MAE 0.36 and RMSE 0.46 (Table 2). Therefore, these descriptors should be kept in mind while designing new molecules that act against EGFR L858R mutant.

### Model for predicting inhibitors against hybrid inhibitors

#### (i) Performance of model on whole hybrid dataset (hybrid_whole)

Finally we have integrated both types of inhibitors (EGFR wild and L858R mutant inhibitors) and developed three hybrid models using only PaDEl descriptors. First we developed a model using all 184 hybrid inhibitors and achieved a correlation of 0.8333/0.7311 (R/R^2^) with MAE and RMSE of 0.49 and 0.63 respectively.

#### (ii) Performance of model on training hybrid dataset (hybrid_train)

Next, we developed model on 147 inhibitors (hybrid_train) using five fold-cross-validation technique and achieved a correlation of 0.85/0.72 (R/R^2^) with MAE and RMSE of 0.46 and 0.63.

#### (iii) Performance of model on validation hybrid dataset (hybrid_valid)

Finally, we evaluate the performance of hybrid_train inhibitors based model on the validation dataset (hybrid_valid) and achieve a correlation coefficient (R)/coefficient of determination (R^2^) of 0.76/0.62 with MAE 0.61 and RMSE 0.72 (Table 2). The purpose of this model is to identify those inhibitors, which are effective against both wild type EGFR, and mutant EGFR.

### SVM implemented using SVM^light^

Besides SMOreg module of Weka we also used SVM^light^ for developing SVM based QSAR models wild, mutant and hybrid type EGFR inhibitors (Table S5 in File S1). First we have developed SVM models for wild_whole, wild_train and wild_valid inhibitors and achieved correlation coefficient (R) of 0.842, 0.850, and 0.845 with MAE and RMSE of 0.445, 0.440, 0.446 and 0.612, 0.606, 0.614 respectively. Next, we have used mutant_whole, mutant_train and mutant_valid datasets and achieved correlation coefficient (R) of 0.762, 0.784, and 0.751 with MAE and RMSE of 0.521, 0.520, 0.530 and 0.638, 0.623, 0.639 respectively. Finally, we have developed SVM models using hybrid_whole, hybrid_train and hybrid_valid inhibitors and achieve a correlation coefficient (R) by 0.814, 0.836, 0.797 with MAE 0.504, 0.495, 0.511 and RMSE 0.667, 0.654, 0.670 respectively. We also developed models for mutant and hybrid inhibitors (Table S5 in File S1).

### Comparision with existing methods

First we compared our wild-EGFR model with that published by Vema A *et al* [23], where they achieved R^2^ of 0.8492 and 0.499 on training and test dataset respectively by using RSA (receptor surface analysis) based model. Next we compared our results with that of Hongying Du *et al*. [16] who have used the same dataset of 128 EGFR inhibitors for developing QSAR models. They achieved (R = 0.918 with R^2^ = 0.843 and MAE = 0.369) using G-PPR based model, while the hybrid model developed in this article achieves (R = 0.921 with R^2^ = 0.847 and MAE = 0.349) on all dataset based QSAR model (Table 4). As there is no other report in the literatures for predicting L858R mutant EGFR inhibitors we cannot compare the results obtained for mutant EGFR.

**Table 4:** Comparative performance of existing method with our method developing for predicting inhibitors against wild type EGFR inhibitors.

Methods	Datasets	R	R^2^	MAE	RMSE
Vema A *et al*	Wild_whole	0.877	0.768	0.434	0.551
	Wild_train	0.922	0.849	0.354	0.435
	Wild_valid	0.730	0.499	0.719	0.846
Hongying Du *et al*	Wild_whole	0.918	0.843	0.369	0.455
	Wild_train	0.921	0.849	0.354	0.442
	Wild_valid	0.901	0.807	0.432	0.504
ntEGFR	Wild_whole	0.921	0.847	0.349	0.450
	Wild_train	0.917	0.837	0.333	0.462
	Wild_valid	0.902	0.810	0.342	0.501

### Cross-prediction

In order to evaluate the cross-suitability of wild and mutant EGFR models we perform cross prediction using both models. First we tested all mutant inhibitors on wild type EGFR model and achieved a correlation coefficient (R)/coefficient of determination (R^2^) 0.3107/0.09644, MAE = 1.1741 and RMSE = 1.4023. Next, we apply reverse strategy where wild type EGFR molecules are tested against the mutant EGFR model and achieved a correlation coefficient (R)/coefficient of determination (R^2^) 0.1674/0.03567, MAE = 1.1189 and RMSE = 1.4062. From this, we conclude that the method trained on wild type EGFR inhibitors is not suitable for prediction of mutant EGFR inhibitors and vice-versa. This may be due to diverse sets of molecules, as all 128 EGFR inhibitors are quinazoline derivatives whereas mutant EGFR inhibitors belong to diverse scaffold particularly imidazothiazoles and pyrazolopyrimidines.

## Discussion

Out of myriad compounds obtained from synthetic processes, the identification of inhibitors, which can serve as lead molecule using experimental techniques is very expensive, time-consuming as well as skill intensive. Thus, it is necessary that new computational methods are developed for shortlisting effective leads. Although in past, some computational methods have been developed for predicting EGFR inhibitors that prevent ATP-EGFR interaction, to the best of authors knowledge, no software/webserver has been developed for predicting EGFR inhibitors. So, in this study, our main goal is to develop an open source webservice for predicting both wild type as well as mutant EGFR inhibitors. First, we identified more frequent fragments present in wild and mutant EGFR inhibitors. These frequent fragments can be used to design the inhibitors of desired activity against wild and mutant EGFR inhibitors. Backbone of any QSAR or prediction model is feature or descriptor of correlation. Though there are numerous software packages in market; our major emphasis was on open source software in order to make these models available for scientific community. As shown in result section, descriptors computed using free software PaDEL that perform as good as any commercial software. Thus in this study we used PaDEL computed descriptors for developing QSAR models. One of the major challenges in QSAR studies is to select relevant or best descriptors that can be used to develop prediction models for predicting inhibitors against wild and mutant EGFR. We developed QSAR models using selected descriptors of wild and mutant EGFR inhibitors by machine learning techniques. In this study, module SMOreg of Weka and SVM^light^ package were used to implement SVM for regression for developing prediction models. First, we developed QSAR models for wild type EGFR inhibitors using selected descriptors of the Dragon, Vlife, PaDEL, WebCDK and PowerMV. We achieved maximum correlation coefficient (R) 0.89 and coefficient of determination (R^2^) 0.83 using PaDEL descriptors by SMOreg. Here, we observed that model developed using PaDEL descriptors perform comparable or better than models developed using other software packages. It was observed that SVM implemented using SMOreg performs better than SVM^light^, for predicting wild type EGFR inhibitors.

Next, we compiled L858R mutant EGFR and developed a SMOreg based prediction model and achieved maximum correlation coefficient (R) 0.83 and coefficient of determination (R^2^) 0.71 while SVM^light^ based model gives 0.76 (R) and 0.56 (R^2^) using PaDEL descriptors. Here, we have observed that the performance of the mutant EGFR model is lower than wild type EGFR that is due to the presence of a diverse set of molecules in the mutant EGFR dataset. Finally, we combined both types of inhibitors and developed a hybrid prediction method. The performance of hybrid based model is lesser than the individual models of wild and mutant EGFR. In case of inhibitors against EGFR mutant also we observed that SVM based models implemented using SMOreg perform bettter than SVM models implemented using SVM^light^. The beauty of this study is that for the first time we have developed a method which would be applicable for both types wild and mutant EGFR inhibitors. We also compared our wild type EGFR based model with existing methods and found that our model gives better results. Based on our optimized models a webserver and computer programs have been designed and developed. This server can be used to identify both wild type EGFR and L858R mutant EGFR inhibitors. In addition, we have integrated analog based inhibitor designing. As the software will be an open source, it is expected that the advancement made in developing this software will be of use and value to the researcher's community working in the field of cancer drug discovery. Also, it is anticipated that the web-services would be highly useful for designing inhibitors for wild as well as mutant EGFR.

## Webserver

We develop a web server “ntEGFR” (available at http://crdd.osdd.net/raghava/ntegfr) using CGI-PERL, PERL and PHP and python scripts using different learning models. The user can paste or upload the structure of the ligand molecule in the server. In this webserver we have provided three types of prediction service, one for wild type EGFR inhibitors, second for L858R mutant EGFR and third for hybrid EGFR inhibitors. Additionally, we also provide analog based inhibitors designing facility in web-service. The current version of ntEGFR is available in three forms; (i) Webserver (ii) Standalone and (iii) Galaxy based server.

## Conclusions

ntEGFR is a open source web server for predicting inhibitory activity (IC_50_) of molecules against wild and mutant EGFR. We have provided three type of prediction models called wild_EGFR, mutant_EGFR and hybrid_EGFR. This web server has three options including; i) prediction of inhibitors against both types of EGFR, ii) screening of large chemical libraries of EGFR inhibitors, iii) generating chemical analogs of EGFR inhibitors.

## Supporting Information

File S1 Table S1, Top 15 selected fragments favoured in wild type EGFR inhibitors. Table S2, Description of some selected chemical descriptors. Table S3, The pair-wise correlation values of 7 docking energy-based descriptors (Wild EGFR). Table S4, Matrix showing the pair-wise correlation values for the 7 descriptors generated by docking (mutant EGFR). Table S5, SVMreg based model evaluation results.
(DOCX)
