# Supplementary material for: QSAR-Based Models for Designing Quinazoline/Imidazothiazoles/Pyrazolopyrimidines Based Inhibitors against Wild and Mutant EGFR
Source: PLoS One. 2014 Jul 3;9(7):e101079. doi: 10.1371/journal.pone.0101079 (PMC4081576; doi:10.1371/journal.pone.0101079)
Supplement: File S1 — Table S1, Top 15 selected fragments favoured in wild type EGFR inhibitors. Table S2, Description of some selected chemical descriptors. Table S3, The pair-wise correlation values of 7 docking energy-based descriptors (Wild EGFR). Table S4, Matrix showing the pair-wise correlation values for the 7 descriptors generated by docking (mutant EGFR). Table S5, SVMreg based model evaluation results. (DOCX) [file pone.0101079.s001.docx]

Supplementary files

QSAR-based models for designing quinazoline/ imidazothiazoles/ pyrazolopyrimidines based inhibitors against wild and mutant EGFR

Jagat Singh Chauhan^1^, Sandeep Kumar Dhanda^1^, Deepak Singla^1^, Open Source Drug Discovery Consortium^2^, Subhash M. Agarwal^3*^ and Gajendra P.S. Raghava^1*^

*Address for Correspondence

Email: [raghava@imtech.res.in](mailto:raghava@imtech.res.in) (GPSR) ; [smagarwal@yahoo.com](mailto:smagarwal@yahoo.com) (SMA)

**Table S1: Top 15 selected fragments favoured in wild type EGFR inhibitors.**

| **Fingerprint key** | **Present in wild EGFR inhibitors** | **Present in mutant EGFR inhibitors** | **Fragment_frequency** |
| --- | --- | --- | --- |
| FP946 | 128 | 1 | 1.426356589 |
| FP311 | 128 | 1 | 1.426356589 |
| FP521 | 128 | 2 | 1.415384615 |
| FP273 | 128 | 2 | 1.415384615 |
| ExtFP120 | 128 | 2 | 1.415384615 |
| ExtFP109 | 128 | 2 | 1.415384615 |
| FP275 | 128 | 3 | 1.404580153 |
| ExtFP780 | 128 | 3 | 1.404580153 |
| ExtFP581 | 128 | 3 | 1.404580153 |
| ExtFP107 | 128 | 3 | 1.404580153 |
| ExtFP911 | 128 | 4 | 1.393939394 |
| FP306 | 128 | 5 | 1.383458647 |
| ExtFP605 | 128 | 5 | 1.383458647 |
| ExtFP256 | 128 | 5 | 1.383458647 |
| FP349 | 128 | 6 | 1.373134328 |

**Table S2: Description of some selected chemical descriptors.**

| **Descriptors Name** | **Category** | **Description** |
| --- | --- | --- |
| FP271, FP313, FP334, FP359, FP421, FP436, FP680, FP946, FP311, FP521, FP273, FP275, FP306, FP349 | CDK fingerprints | Fingerprint of length 1024 and search depth of 8, Path based, hashed fingerprint. |
| ExtFP121, ExtFP471, ExtFP668, ExtFP678, ExtFP914 , ExtFP120, ExtFP109, ExtFP780, ExtFP581, ExtFP107, ExtFP911, ExtFP605, ExtFP256 | CDK extended fingerprints | Extended Fingerprint (ExtFP, 1024 bits)  Extends the FP with additional bits describing ring features. |
| KRFP1931 | Klekota-Roth fingerprins | Presence of chemical substructures.  (KRFP, 4860 bits) |
| GraphFP136 | CDK graph only fingerprint | Specialized version of the Fingerprinter which does not take bond orders into account |
| SssNHcount | physicochemical descriptors | SssNHcount is a descriptor defines the total number of –NH group connected with two single bonds, indicates a negative contribution to the biologic activity |
| SaaOcount | physicochemical descriptors | Rotatable bonds and aromatic oxygen (SaaOcount)  This descriptor signifies total number o f oxygen connected with two aromatic bonds. |
| L2u c.026, | WHIM directional descriptors  Transition of Polarity(2) | 2nd component size directional WHIM index / unweighted |
| B04.N.O.,  B10.C.Br,  Csp3_05_Osp3  Nsp2_06_Osp2  xVDW_EN | 2D binary atom pairs of order 4  2D binary atom pairs of order 10  Geometry Optimization  Geometry Optimization  Potential Energy Descriptors | Presence/absence of N - O at topological distance 4  Presence/absence of C - Br at topological dis tance 10  Carbon and Oxygen atom sp3  van der Waals component of the potential energy. xVDW is the van der Waals radii of the halogen bond don or atom |
| minaaN | Electro-topological StateAtom Type | Descriptor with Minimum atom-type E-State: :NH: |

Table S3: The pair-wise correlation values of **7 docking energy-based descriptors (wild type EGFR).**

| **Descriptors** | **E_FreeBind_** | **EI_nterMol_** | **E_VHD_** | **E_Elec_** | **E_FToT_** | **E_Tors_** | **E_Unb_** |
| --- | --- | --- | --- | --- | --- | --- | --- |
| E_FreeBind_ | 1.000 | **0.728** | **0.603** | 0.447 | 0.393 | -0.348 | -0.197 |
| E_InterMol_ | **0.728** | 1.000 | **0.934** | 0.320 | 0.452 | -0.801 | 0.209 |
| E_VHD_ | **0.603** | 0.934 | 1.000 | -0.033 | 0.445 | -0.811 | 0.280 |
| E_Elec_ | 0.447 | 0.320 | -0.033 | 1.000 | 0.086 | -0.104 | -0.158 |
| E_FToT_ | 0.393 | 0.452 | 0.445 | 0.086 | 1.000 | -0.716 | 0.492 |
| E_Tors_ | -0.348 | -0.801 | -0.811 | -0.104 | -0.716 | 1.000 | -0.440 |
| E_Unb_ | -0.197 | 0.209 | 0.280 | -0.158 | 0.492 | -0.440 | 1.000 |

Table S4: Matrix showing the pair-wise correlation values for the 7 descriptors generated by docking (mutant EGFR).

| Descriptors | E_FreeBind_ | EI_nterMol_ | E_VHD_ | E_Elec_ | E_FToT_ | E_Tors_ | E^Unb^ |
| --- | --- | --- | --- | --- | --- | --- | --- |
| E_FreeBind_ | 1.0000 | 0.8054 | 0.8023 | -0.0066 | 0.5493 | 0.2024 | -0.0156 |
| E_InterMol_ | 0.8054 | 1.0000 | 0.8939 | 0.2141 | 0.0874 | -0.0813 | -0.0092 |
| E_VHD_ | 0.8023 | 0.8939 | 1.0000 | -0.2463 | 0.2851 | -0.1169 | 0.1254 |
| E_Elec_ | -0.0066 | 0.2141 | -0.2463 | 1.0000 | -0.4312 | 0.07797 | -0.2932 |
| E_FToT_ | 0.5493 | 0.0874 | 0.2851 | -0.4312 | 1.0000 | -0.0743 | 0.4017 |
| E_Tors_ | 0.2024 | -0.0813 | -0.1169 | 0.0779 | -0.0743 | 1.0000 | -0.1907 |
| E_Unb_ | -0.0156 | -0.0092 | 0.1254 | -0.2932 | 0.4017 | -0.1907 | 1.0000 |

Table S5. SVMreg based model evaluation results.

| **Inhibitors** | **Descriptors** | **R** | **R^2^** | **MAE** | **RMSE** |
| --- | --- | --- | --- | --- | --- |
| Wild EGFR | wild_whole (128 inhibitors) | 0.842 | 0.711 | 0.445 | 0.612 |
|  | wild_train (103 inhibitors) | 0.850 | 0.719 | 0.440 | 0.606 |
|  | wild_valid (25 inhibitors) | 0.845 | 0.712 | 0.446 | 0.614 |
| Mutant EGFR (L858R) | mutant_whole (56 inhibitors) | 0.762 | 0.569 | 0.521 | 0.638 |
|  | mutant_train (42 inhibitors) | 0.784 | 0.586 | 0.520 | 0.623 |
|  | mutant_valid(14 inhibitors) | 0.751 | 0.564 | 0.530 | 0.639 |
| Hybrid | hybrid_whole (184inhibitors) | 0.814 | 0.662 | 0.504 | 0.667 |
|  | hybrid_train(147 inhibitors) | 0.836 | 0.694 | 0.495 | 0.654 |
|  | hybrid_valid (37 inhibitors) | 0.797 | 0.659 | 0.511 | 0.670 |
